# Gamma-Normal-Gamma Mixture Model for Detecting Differentially Methylated Loci in Three Breast Cancer Cell Lines

**Published:** 2007-02-07

**Authors:** Abbas Khalili, Dustin Potter, Pearlly Yan, Lang Li, Joe Gray, Tim Huang, Shili Lin

**Affiliations:** 1 Department of Statistics, The Ohio State University, Columbus, OH 43210; 2 Human Cancer Genetics, The Ohio State University, Columbus, OH 43210; 3 Division of Biostatistics, Department of Medicine, Indiana University School of Medicine, One Cyclotron Rd. Indianapolis, IN 47405; 4 Lawrence Berkeley National Laboratory, Berkeley, CA 94720; 5 Mathematical Biosciences Institute, The Ohio State University, Columbus, OH 43210

**Keywords:** CpG islands, mixture modeling, methylation/epigenetic signature, microarrays, breast cancer cell lines

## Abstract

With state-of-the-art microarray technologies now available for whole genome CpG island (CGI) methylation profiling, there is a need to develop statistical models that are specifically geared toward the analysis of such data. In this article, we propose a Gamma-Normal-Gamma (GNG) mixture model for describing three groups of CGI loci: hypomethylated, undifferentiated, and hypermethylated, from a single methylation microarray. This model was applied to study the methylation signatures of three breast cancer cell lines: MCF7, T47D, and MDAMB361. Biologically interesting and interpretable results are obtained, which highlights the heterogeneity nature of the three cell lines. This underlies the premise for the need of analyzing each of the microarray slides individually as opposed to pooling them together for a single analysis. Our comparisons with the fitted densities from the Normal-Uniform (NU) mixture model in the literature proposed for gene expression analysis show an improved goodness of fit of the GNG model over the NU model. Although the GNG model was proposed in the context of single-slide methylation analysis, it can be readily adapted to analyze multi-slide methylation data as well as other types of microarray data.

## 1. Introduction

Mammalian DNA methylation occurs when a methyl group is added to the cytosine residue of a CpG dinucelotide (Jaenisch and Bird, 2003) and indeed a large portion of genomic DNA is methylated in CpG dinucleotides. Essential exceptions are the CpG islands (CGIs) within gene promoters that are generally unmethylated in normal cells. For more than two decades it has been known that DNA methylation is associated with gene activity and chromatin structure. Recent studies have demonstrated that aberrant CpG methylation is connected to human diseases such as cancer (Laird, 2005; Yang et al. 2006; Wang et al. 2006), Niemann-Pick Disease (Simonaro et al. 2006), lupus (Ballestar et al. 2006; Patole et al. 2005), and Rett syndrome (Kriaucionis and Bird, 2003).

Implementation of state-of-the-art microarray technologies has made possible the measurement of the methylation signature of multiple genes simultaneously (Piotrowski et al. 2006; Shen et al. 2006; Yan et al. 2002). Methylation signatures, and more generally epigenetic signatures, play an important role in furthering the system-wide understanding of the biological mechanism of disease. Recently, groups have conceptualized, and in a small part realized, the idea of a human epigenome project (Jones and Martienssen, 2005; Rakyan et al. 2004). As the human genome project has advanced discoveries in both basic and translational science, it is expected that the human epigenome project combined with high-throughput methylation assays will facilitate our fundamental understanding of aberrant epigenetic mechanisms.

Two aspects of microarray data make the analysis exceptionally challenging. First, even with recent advances in the microarray technology, there exists experimental variability, which makes detection of genuine biological differences dificult to accomplish as the two may be confounded (Verducci et al. 2006). Second, if p genes are measured in the microarray experiments, then the microarray data represents a p-dimensional random vector with mutually dependent components. Cost, both monetary and temporal, make it inhibitive to generate more than a small number (with respect to p) of replicates. This “large-p-small-n” scenario prevents the use of classical statistical approaches to data analysis (Verducci et al. 2006).

In some circumstances microarray analysis is further hampered by the lack of any experimental or biological replicates. Note that a single-slide analysis (n = 1), when there is no replication at all, is the “large-p-small-n” scenario taken to the extreme. This may be due to lack of resources (such as time, biological samples, arrays, etc.) for a larger experimental study. For example, a pilot study may be interested in the epigenetic differences between tumors in different stages of progression; however, due to limited financial resources only one sample from each stage is assayed. Once the human epigenome project is completed, another conceivable situation is the clinical application of high-throughput arrays for assaying the methylation signature of DNA markers associated with different diseases. Gene expression data from microarrays has recently been used to model interactions of multiple risk factors associated with clinical outcome for use in individual patient care (Potti et al. 2006; Li, 2006; Pittman et al. 2004). The integration of genome wide methylation information from a single slide patient sample will potentially enhance and strengthen the clinical predictive power of such risk models (West et al. 2006).

A number of methods have been suggested for finding genes that are differentially expressed between two variants for single-slide data, all of which are proposed for gene expression microarray data. The most common approach is to flag those with an intensity ratio above a given cut-off, typically between 1.5 and 3. Such a selection criterion is ad-hoc in nature. More formal statistical treatments are available in the literature. Newton et al. (2001) suggested modeling the Cy5 and Cy3 signals on an array as independent random variables each following a gamma distribution. Such an approach benefits from considering the thousands of measurements on an array simultaneously rather than individually (Efron and Morris, 1973; Carlin and Louis, 1996). A different single-slide applicable approach was recently proposed by Dean and Raftery (2005), which models the normalized log intensity ratios as coming from a two-component, Normal and Uniform (NU), mixture of distributions.

In this paper, we present a methodology for the detection of differentially methylated loci between the two samples co-hybridized onto a methylation microarray. The log ratio of the Cy5 to Cy3 intensities of the probes on the array are modeled as random variables sampled from one of three distributions. These three groups are biologically interpreted as hypomethylated: the assayed DNA from the Cy3 sample is methylated while the Cy5 sample is not; hypermethylated: the Cy5 sample is methylated and the Cy3 sample is not; and undifferentiated: there is no observable difference in methylation status between the two samples. This model was applied to three breast cancer cell lines, MCF7, T47D, and MDAMB361. These analyses yielded biologically meaningful results, which are consistent with the current understanding of the role of aberrant methylation in breast cancer development. A boot strap resampling technique was used to evaluate the reliability of the probe classifications from the fitted model. We further assessed the goodness of fit of this model by comparing it to that of the NU model (Dean and Raftery, 2005) based on the Kullback-Leibler distance measure.

## 2. Methods

In this section we first introduce a mixture model for identifying differentially methylated loci (probes) in the sample. An algorithm is described for estimating the parameters of the model. Then, we outline the usage of the model for classifying the probes into the three groups: hypomethylated, undifferentiated, and hypermethylated.

### 2.1. Gamma-Normal-Gamma mixture model

Consider the Red (R; Cy5) and Green (G; Cy3) intensity measurements. Our interest is to model *Y*, the normalized log ratio, log_2_(*R/G*), by the Gamma-Normal-Gamma (GNG) model. Our assumption here is that certain steps have already been taken to normalize the data first. Briefly, loess normalization transform as described in (Yang et al. 2002) is applied to the data, however, the loess fit is based on a rank-invariant subset as in (Speed, 2003).

We begin by modeling probes as three different groups: hypomethylated, undifferentiated, and hypermethylated. Since the undifferentiated probes have a true log ratio of zero, we model the observed log ratio for these probes by a Normal distribution. For the hypermethylated probes, the log ratio will be positive and hence a Gamma distribution will be a reasonable choice for modeling this group of probes. The choice of the Gamma model is mainly due to the perception that the characteristics of the distribution match well with the biological data it models. In fact, the Gamma model has a long history in statistical ecology (e.g. Fisher et al. 1943) due to both its analytical convenience and its possession of a potential deeper biological interpretation, and it has recently been used in microrray analysis (Newton et al. 2001). The log ratios corresponding to the hypomethylated probes are negative, and we choose to use the mirror image of the Gamma distribution used for the hypermethylated probes for this group of probes. However, we choose a different set of parameters for the mirror image Gamma distribution. Thus, overall, a mixture of three distributions, that is, one Normal and two different Gamma distributions will be used to model the normalized log ratio *Y*. Mathematically, the model is given as follows.

Consider the indicator function *I*_{*y* > 0}_ = 1, for *y* > 0, and zero otherwise. Our proposed mixture model is then given by

(1) $$\begin{array}{l} f\;(y;\Psi )=\pi_{1}G(-y;\alpha_{1},\beta_{1})I_{\left\{-y>0\right\}}\\ \;+\pi_{2}\;N(y;\;\mu ,\;\sigma^{2})\\ \;+\pi_{3}\;G(y;\alpha_{2},\beta_{2})I_{\left\{y>0\right\}} \end{array}$$

where π*_k_* > 0, *k* = 1, 2, 3, are called mixing proportions, and they represent the proportion of each of the three above mentioned groups, and further $\sum_{k=1}^{3}\pi_{k}=1$. The *G*(·; α, β) and *N*(·; μ, σ^2^) stand for the Gamma and Normal density functions, and

$$\Psi =(\alpha_{1},\;\beta_{1},\;\alpha_{2},\;\beta_{2},\;\mu ,\;\sigma^{2},\;\pi_{1},\;\pi_{2},\;\pi_{3})$$

is the vector of all unknown parameters in the model, but note that the π’s are interdependent. In (1), the π_1_ and π_3_ show the probabilities of each probe being hypomethylated and hypermethylated, respectively. The π_2_ is the probability that a probe is not differentially methylated. Although the theoretical mean of the normal component is zero, we opted to allow this value to be estimated jointly with the other parameters to capture any residual bias that has not been removed by normalization.

### 2.2. Parameter estimation

Since our modeling approach is fully parametric, we use the most popular parameter estimation method, that is maximum likelihood estimation, for estimating the parameter vector Ψ.

Let *y_i_*, *i* = 1, 2, ..., *n*, be the normalized log ratios. The log-likelihood function of the parameter vector Ψ is given by

$$l_{n}\left(\Psi \right)=\sum_{i=1}^{n}\text{log}\;f\;\left(y_{i};\Psi \right)$$

where *f* (*y_i_*; Ψ) is the mixture density function in (1). The Maximum Likelihood Estimate (MLE) of Ψ is then given by

$${\hat{\Psi }}_{n}=\text{arg}\underset{\Psi }{\text{max}}l_{n}\left(\Psi \right).$$

Due to the complexity of the log-likelihood function *l_n_*(Ψ), there is no apparent analytical form for the estimator **Ψ̂***_n_*. Thus a numerical method is needed to find the solution to the above maximization problem. In the context of mixture models, the Expectation-Maximization (EM) algorithm of Dempster, Larid and Rubin (1997) provides a convenient approach to obtain the MLE **Ψ̂***_n_*. The EM algorithm in our problem is outlined as follows.

Let *z_ik_*, *k* = 1, 2, 3, be indicator variables showing the component membership of each observation *y_i_* in the mixture model (1). Note that *z_ik_*’s are unobservable (missing) variables. The EM algorithm works with the complete data log-likelihood function

$$\begin{array}{l} l_{n}^{c}\left(\Psi \right)=\sum_{i=1}^{n}\sum_{k=1}^{3}z_{ik}\text{log}\pi_{k}\\ \;+\sum_{i=1}^{n}\{z_{il}\text{log}\left[G\left(-y_{i};\alpha_{1},\beta_{1}\right)\right]I_{\left\{-y_{i}>0\right\}}\\ \;+z_{i2}\text{log}\left[N\left(y_{i};\mu ,\sigma^{2}\right)\right]\\ \;+z_{i3}\text{log}\left[G\left(y_{i};\alpha_{2},\beta_{2}\right)\right]I_{\left\{y_{i}>0\right\}}\}. \end{array}$$

The EM algorithm finds **Ψ̂***_n_* iteratively in two steps as follows.

**E-Step:** Let Ψ^(^*^m^*^)^ be the estimate of Ψ after the *mth* iteration. The E-step computes the conditional expectation of the function $l_{n}^{c}$(Ψ) with respect to *P* (***z*** | ***y***, Ψ^(^*^m^*^)^), where ***z*** = {*z_ik_*, *i* = 1, …, *n*, *k* = 1, 2, 3.}, and ***y*** = {*y*_1_, …, *y_n_*}. The conditional expectation is found to be

$$\begin{array}{l} Q\left(\Psi ;\Psi^{\left(m\right)}\right)=\sum_{i=1}^{n}\sum_{k=1}^{3}w_{ik}^{\left(m\right)}\text{log}\pi_{k}\\ \;+\sum_{i=1}^{n}\{w_{i1}^{\left(m\right)}\text{log}\left[G(-y_{i};\alpha_{1},\beta_{1})\right]I_{\left\{-y_{i}>0\right\}}\\ \;+w_{i2}^{\left(m\right)}\text{log}N(y_{i};\mu ,\sigma^{2})\\ \;+w_{i3}^{\left(m\right)}\text{log}\left[G(y_{i};\alpha_{2},\beta_{2})\right]I_{\left\{y_{i}>0\right\}}\}. \end{array}$$

where

$$\begin{array}{l} w_{i1}^{\left(m\right)}=\frac{\pi_{1}^{\left(m\right)}G\left(-y_{i};\alpha_{1}^{\left(m\right)},\beta_{1}^{\left(m\right)}\right)I_{\left\{-y_{i}>0\right\}}}{f\;(y_{i};\Psi^{\left(m\right)})},\\ w_{i2}^{\left(m\right)}=\frac{\pi_{2}^{\left(m\right)}N(y_{i};\mu^{\left(m\right)},\sigma^{2(m)})}{f\;(y_{i};\Psi^{\left(m\right)})},\\ w_{i3}^{\left(m\right)}=\frac{\pi_{3}^{\left(m\right)}G\left(y_{i};\alpha_{2}^{\left(m\right)},\beta_{2}^{\left(m\right)}\right)I_{\left\{y_{i}>0\right\}}}{f\;(y_{i};\Psi^{\left(m\right)})},i=1,\ldots ,n, \end{array}$$

are the conditional expectations of the *z_ik_*’s.

**M-Step:** The M-step on the (*m+1*)*th* iteration maximizes the function *Q*(Ψ; Ψ^(^*^m^*^)^) with respect to Ψ. By maximizing this function with respect to π*_k_*’s, μ and σ^2^, we have

$$\begin{array}{l} \pi_{k}^{\left(m+1\right)}=\frac{1}{n}\sum_{i=1}^{n}w_{ik}^{\left(m\right)},k=1,2,3,\\ \mu^{\left(m+1\right)}=\frac{\sum_{i=1}^{n}w_{i2}^{\left(m\right)}y_{i}}{\sum_{i=1}^{n}w_{i2}^{\left(m\right)}},\\ \sigma^{2(m+1)}=\frac{\sum_{i=1}^{n}w_{i2}^{\left(m\right)}{\left(y_{i}-\bar{y}\right)}^{2}}{\sum_{i=1}^{n}w_{i2}^{\left(m\right)}}. \end{array}$$

Maximization with respect to (α*_k_*, β*_k_*), *k* = 1, 2, needs to be done by using a numerical method. In the current study we used the function *optim*( ) in R for this task. This function implements the method of Nelder and Mead (1965) to maximize the objective function.

Starting from an initial value Ψ^(0)^, the EM algorithm iterates between the E and M-steps until some convergence criterion is satisfied. For example, for a pre-specified value ɛ > 0, the algorithm will stop if

$$\left\|\Psi^{\left(m+1\right)}-\Psi^{\left(m\right)}\right\|<\varepsilon .$$

This was the criterion used in the analyses carried out in this paper, with ε = 10^−12^ and the use of the *L*^2^ norm.

Due to the sensitivity of the results to the choice of starting point, it is recommended that multiple sets of random and non-random initial parameter values be used to increase the chance of finding the global maximum, especially if the likelihood surface is multimodal. For our applications, we used 20 sets of starting values; the estimates that led to the maximum likelihood were taken as our MLE.

### 2.3. Classification

Finite mixture models provide a model-based approach for classification (McLachlan and Peel, 2000). Consider a finite mixture model with *K* components which in fact corresponds to *K* classes. In the model-based classification one first calculates the posterior probability that an observation belongs to any of the classes *k* = 1, 2, ..., *K*. Then the observation is classified into the class with highest posterior probability. Accordingly, the classification based on the GNG mixture model can be performed as follows.

Consider the log ratios *Y_i_*, *i* = 1, 2, ..., *n*, that are assumed to follow the GNG mixture model in (1). The posterior probabilities that *Y_i_* belongs to any of the classes 1, 2 or 3 are given by

$$\begin{array}{l} p_{i1}=P(Y_{i}\in 1|Y_{i}=y_{i})=\frac{\pi_{1}G(-y_{i};\alpha_{1},\beta_{1})I_{\left\{-y_{i}>0\right\}}}{f\;(y_{i};\Psi )},\\ p_{i2}=\text{P}(Y_{i}\in 2|Y_{i}=y_{i})=\frac{\pi_{2}\;N(y_{i};\mu ,\sigma^{2})}{f\;(y_{i};\Psi )},\\ p_{i3}=P(Y_{i}\in 3|Y_{i}=y_{i})=\frac{\pi_{3}G(y_{i};\alpha_{2},\beta_{2})I_{\left\{y_{i}\;>\;0\right\}}}{f\;(y_{i};\Psi )}. \end{array}$$

Using the MLE **Ψ̂***_n_*, let *p̂_ik_*, *k* = 1, 2, 3, be the MLE’s of the posterior probabilities. A probe with log ratio *Y_i_* is classified to class *k* if

$${\hat{p}}_{ik,max}=\text{max}\{{\hat{p}}_{i1,}{\hat{p}}_{i2},{\hat{p}}_{i3}\}.$$

To increase one’s confidence on the probes classified, especially those labeled as differentially methylated, and to guard against false positives, we decided to classify a probe with log ratio *Y_i_* to class *k* only if

$$\frac{{\hat{p}}_{ik,max}}{{\hat{p}}_{ij}}>C$$

for some constant value *C ≥* 1, for all *j* ≠ *k*; otherwise that particular probe is left as unclassified. We suggest to set the value of *C* to be inversely related to the confidence of the technology generating the data. That is, a small value for *C* should correspond to a high confidence in the sensitivity and reproducibility of the microarray measurement. In the applications to the three cell lines, a value of 2 was chosen for *C* to reflect our degree of confidence in the microarray experiments, which was also partly motivated by ease of interpretation: a two-fold difference in probabilities was required to determine classification. In the situation where a priori information about the quality of the microarray measurement is unavailable, a bootstrap technique as described below may be used as a means to select an appropriate *C* that will lead to high consistency rates.

## 3. Results

### 3.1. Breast cancer data

#### Three cell lines

Breast cancer cell lines have been used as research models for a long time. A large number of them were established in the late 1970s. Two of the cell lines we chose to analyze, MCF7 and T47D, are among the most often studied breast cancer cell lines. They share distinctive features such as isolated originally from pleural effusions, little to no invasiveness, hormone-receptor (estrogen-and progesterone receptor) positive, Her-2/neu negative, expression of *WNT7B* oncogene, and high expression of genes associated with the luminal epithelial-like phenotype. Although there are many similarities between these two cell lines, there also exist differences. First, the age of the patients from which these two cell lines derived from were 69 and 54 for MCF7 and T47D, respectively. As DNA methylation increases with age, we would expect more methylation events in samples from older patients. Second, though both of these cell lines express estrogen receptor, it was noted by Karey and Sirbasku (1988) that they responded differently to the challenge of 17 beta-estradiol. Third, due to known (caspase 3 is expression in T47D but not in MCF7) and unknown gene expression profiles associated with the cell lines, they were shown to respond differently to gene perturbation (Yamashita et al. 2003) and to drug- and chemical treatments (Green, 2003; Bhat, 2001). Although the MDA-MB-361 cell line shares only one common feature with the other two cell lines (positive hormone receptor status) and has many distinct features (isolated originally from a brain metastasis of a 40 years old patient, moderate in vitro invasiveness, Her-2/neu overexpression, and expression of *WNT7H* + oncogene), it still tends to be closer to the luminal epithelial-like MCF7 and T47D than to the mesenchymal-like breast cancer cell lines. In all, we expect these three cell lines to share some commonly methylated loci and harbor aberrant methylation in loci that were uniquely theirs.

#### Generation of methylation profiles

The array employed to generate the methylation profiles for the three cell lines was custom designed to assay the promoter regions located within a CpG island (regions of the DNA with higher than expected CpG dinucleotides). The oligonucleotide probes printed on the array were selected from a larger library of probes comprising Agilent’s location analysis array (Boyer et al. 2005). Oligonucleotide probes were considered if their target was located within the predicted promoter region of a gene. This subset was further reduced so that the resulting array contained 40659 unique probes with targets located within the predicted promoter region of 10834 unique genes. For each cell line, DNA was isolated from the cells, digested by the restriction enzyme *Bfa*I in order to reduce genomic complexity, and ligand linked for subsequent PCR. Methylation sensitive enzymes, *Hpa*II and *HinP* I1, were used to interrogate the methylation status of the DNA fragments. The remaining intact DNA was then PCR amplified, dye coupled with either Cy5 (cancer samples) or Cy3 (normal samples), and hybridized onto the CpG promoter methylation array as described above. Thus, we have a total of three slides, each captures the methylation signature of a particular cell line. In all three slides, the normal samples come from the immortalized normal breast epithelial cell line MCF10A.

#### Data preprocessing

**Figure 1.**
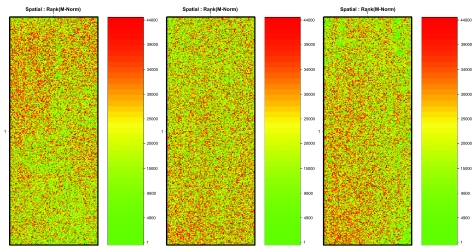
Heat map for the normalized log-ratio of the Cy5 to Cy3 intensity for the three analyzed data sets. Color scheme represents relative ranking of the log-ratio with green denoting a low ranking, or relatively small ratio, and red denoting a high ranking, or relatively large ratio. The x and y axis of the plots denote the position of the probe on the microarray.

**Figure 2.**
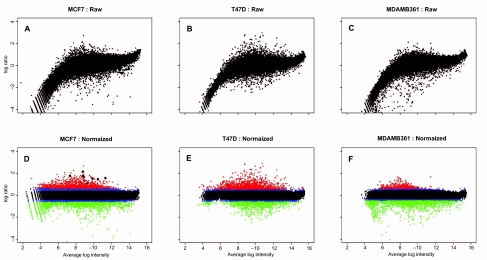
Scatter plots for the average log intensity versus the log ratio for each of the three data sets. A–C: The un-normalized data is plotted. D–F: The loess normalized data is plotted with the hyper- and hypomethylated probes highlighted in red and green, respectively. The unclassified probes are highlighted in blue. The black bullets in D are some of the genes that we had validated as hypermethylated.

By utilizing the ink jet technology, Agilent has eliminated spatial effect often found correlated with print-tip regions on other oligo arrays. Upon inspection of the heat map for the normalized log-ratio of the probes with respect to their position on the array, see Figure 1, no obvious spatial effects were apparent. Therefore, no spatial correction was made. However, Figure 2, A–C show a significant, yet typical, dye effect, and thus the data were normalized as described above. Since the spread of the resulting mean-corrected log-ratios was not significant (0.33 ± 0.03), no methods for adjusting the spread of the data was used (see Figure 2, D–F).

### 3.2. Model fitting

**Figure 3.**
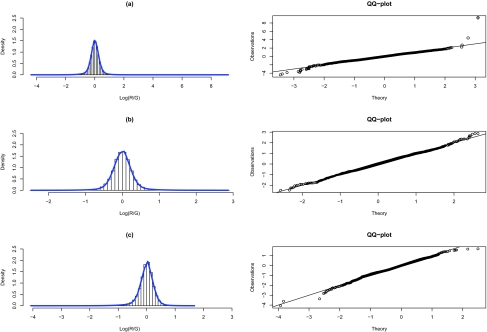
Density plots and QQ plots of fitted model. A–C: density plots of fitted model superimposed on observed data histograms for each of the three datasets. D–F: QQ-plots of the fitted model and the observed empirical distribution, for each of the three datasets

We fitted the GNG model to the normalized data form the three microarray experiments. The parameter estimates corresponding to each experiment are given in Table 1. For all three cell lines, the mixing proportions follow approximately a 15-70-15% split, although deviations among one another is obvious. Similar observations apply to the estimates of the other parameters. In particular, we note that the mean estimates for the normal component are all close to 0, as one would expect with the normalized data, but the three estimates deviate from one another. To check the performance of the fitted GNG model empirically, we plotted the density function of the fitted models over the histograms of the normalized log ratios. Furthermore, QQ-plots are also provided. As can be seen from Figure 3, apart from a few points at the end of the QQ plots not falling right on the straight lines, the plots show that the GNG model provided reasonably good fits to each of the microarray data. Using the classification criterion discussed in 2.3, probes were classified as hypermethylated (red dots in Figure 2, D–F), undifferentiated (black dots), hypomethylated (green dots), or left unclassified (blue dots). This resulted in a total of 965, 1051, and 375 genes, in MCF7, T47D, and MDAMB361, respectively, labeled as hypermethylated, the group that we will focus on in discussing the biological relevance in the next sub-section.

Despite the fact that we know there are three groups of probes: hypomethylated, hyper-methylated and undifferentiated, one may also consider fitting the NU model (Dean and Raftery, 2005), which was proposed to identify differentially expressed genes from non-differentially expressed ones. To compare the relative goodness of fits of the GNG and the NU models to each of the dataset, we use the Kullback-Leibler (K-L) distance. In probability and information theory, the Kullback-Leibler distance (or relative entropy) is a natural distance measure between two probability distributions with densities functions, say, *f* (*x*) and *g* (*x*), and is defined as

(2) $$KL(f,g)=\int_{-\infty }^{+\infty }f\;(y)\text{log}\frac{f\;(y)}{g(y)}\;dy.$$

The K-L distance is always non-negative, and *KL*(*f, g*) = 0 if and only if *f* = *g*. In applications, typically *f* represents the empirical distribution of the observed data, and *g* represents a proposed approximation of *f*. In the current application, the proposed GNG or the NU mixture plays the role of *g*, after the fitted density is discretized, and thus (2) becomes a summation. Table 2 shows the K-L distance based on each of the GNG and NU mixture models as well as a measure of reduction of distance, for each the three experiments under consideration. As we can see from the table, the KL distances are much larger under the NU model than under the GNG model.

We further investigated the reliability of the GNG model for classification using a boot strap resampling technique. For each of the three experiments under study, we generated a bootstrap sample in each experiment and re-classified the probes flagged in the original data based on the model fitted with the bootstrap sample. We then calculated the proportion of the hypermethylated loci that retained the same classification as the originals, and referred to it as the rate of consistency. We generated *B* = 200 bootstrap samples for each of the three experiments, which resulted in a mean consistency rate of 94.6, 98.4, and 99.0%, respectively.

### 3.3. Biological relevance of hypermethylated genes

For the given data set, the GNG model identifies genes for which their promoter regions are hyper-as well as hypomethylated in the breast cancer cell lines. However, as our research focus is on genes down-regulated by DNA methylation, we will confine our discussion concerning the biological relevance of the target genes only to the subset of hypermethylated genes.

**Figure 4.**
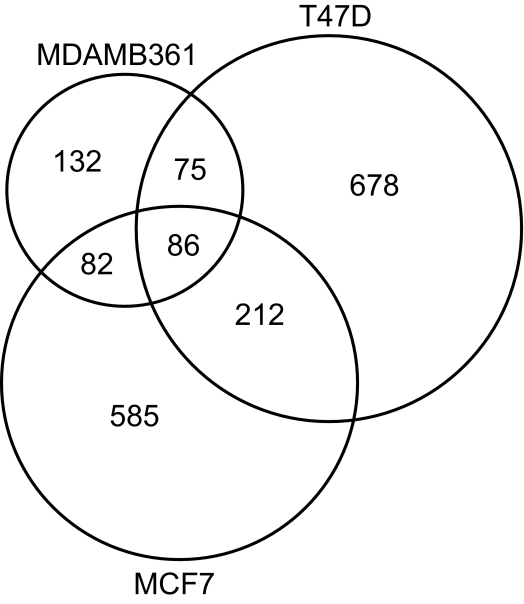
Venn diagram denoting the intersection between the sets of hypermethylated genes for each of the three experiments. The radius of each circle is relative to the number of genes in each set.

Cancer cell lines are known to have more methylation events than primary tumors due to the effect of long-term culturing of DNA methylation. This is evident in the number of methylated genes identified by the GNG model in the breast cancer cell lines. T47D has many methylated genes (1,051), followed closely by MCF7 (965), and MDA-MB-361 has significantly less methylated targets (375). This is equivalent to about 4–10% methylation as our microarray platform contains close to 11,000 unique genes. In primary breast tumors, we expect to detect around 1–2% methylation events. Of the genes flagged to be hypermethylated, 86 of them are common in all three cell lines. The Venn diagram in Figure 4 also shows that MCF7 and T47D share 212 additional hypermethylated genes, which more than doubles that shared by MDAMB361 and either of these two cell lines. This finding is consistent with our discussion of the characteristics of the cell lines earlier in Section 3.1.

The Gene ontology tree machine (GOTM) Zhang et al. (2004) was used to conduct a statistical analysis of the GO terms enriched by the 86 commonly methylated genes. Using the entire genome as the reference population, the category “Sequence-specific DNA binding” is enriched in this set (p-value = 0.0019). Of the seven genes (*HOXB13, FALZ, FOXO3A, GATA2, HOXB9, HOXD11,* and *TERT*) present in this category, a couple of them deserve more detailed discussion. *HOXB13* is a gene we have studied extensively before (Rodriguez et al. 2006). This gene encodes a transcription factor that belongs to the homeobox gene family. It was evaluated through quantitative methylation-specific PCR (qMSP) that higher level of methylation occurred in hormone-receptor positive primary breast tumors, corroborating our current microarray finding. Another sequence-specific DNA binding gene identified is *FALZ*. The protein coded by *FALZ* is highly similar to the largest subunit of the Drosophila *NURF* (nucleosome remodeling factor) complex which interacts with sequence-specific transcription factors to facilitate gene transcription. There is evidence that human *NURF* complex can act as a negative regulator of the JAK/STAT (cytokine signaling) pathway thereby decreasing growth rate and anchorage-independent growth of cancer cells. As such, hypermethylation of *FALZ* can confer growth advantage to these cell lines in many diverse ways. Another member of the sequence-specific DNA binding gene list, *FOXO3A*, belongs to the fork-head family of transcription factors. This gene likely functions as a trigger for apoptosis through expression of genes necessary for cell death. Therefore, for cell lines such as T47D that lacks caspase 3 (Radisavljevic, 2003), the FOXO3a kinase pathway provides a much need means to trigger cellular apoptosis.

In addition to the GO analysis, we decided to verify the methylation status of some of the other genes. Genes were first checked against the literature to establish that their suppression might play a role in cancer development and progression. Then a subset of genes with probes on *BfaI* fragment(s) that overlapped promoter CpG island were selected for validation by methylation-specific PCR in MCF7 cell lines (MSP) (Herman et al. 1996). Genes shown by MSP to be methylated are identified in Figure 3D as brown bullets. It is evident from the plot that the methylation levels of these gene targets varied from moderately methylated to highly methylated.

Yet another approach to evaluate hypermethylated genes identified by the GNG model is to seek out genes evaluated in other breast cancer studies. One such gene is *LATS1*. The GNG model identified this gene to be methylated in all three cell lines studied. Experimental evidence suggests that this gene is a tumor suppressor gene capable of modulating cell survival and negatively regulating cell proliferation by modulating CDC2/Cyclin A activity (Xia et al. 2002). Another gene found to be hypermethylated in MDA-MB-361 and T47D is *ETV5*, also known as *ERM. ETV5* is a member of the superfamily of Ets-related transcription factors. Members of this family bind to related DNA sequence elements in the promoters of target genes to regulate gene transcription. Candidate target genes include ERBB2 (encodes Her2/neu) and genes encode matrix proteases (Bieche et al. 2004). There fore, dysregulation of *ETV5* (by DNA methylation) would lead to increase invasive potential of tumor cells. The observation of promoter hypermethylation in this gene in MDAMB-361 is reasonable as this cell line overexpresses Her2/neu. Such observation in T47D but not in MCF7 is also reasonable as tumors occurring at a younger age (T47D originated from a 54 years old patient) tends to be more aggressive than tumors occurring at an older age (MCF7 originated from a 69 years old patient). Contrarily to *ETV5*, *WT1* was found to be hypermethylated in MCF7 and T47D but not in MDA-MB-361. The *WT1* gene was originally identified as a tumor suppressor gene responsible for Wilms’ tumor, a kidney neoplasm of childhood. Recent findings point to an oncogenic effect of *WT1* in tumor types such as leukemia and breast cancer. In the breast cancer study, (Miyoshi et al. 2002) observed that a high expression of *WT1* conferred a poor prognosis for breast cancer patients independent of other conventional prognostic factors. Therefore, it is reasonable to observe methylation of WT1 promoter in the less aggressive MCF7 and T47D but not in the more aggressive MDA-MB-361 cell line.

## 4. Discussion

In this paper, we proposed a single slide data analysis model (GNG mixture model) for identifying hypermethylated CpG islands loci based on methylation microarray data, and applied it to analyze data generated from three estrogen receptor-positive cell lines. From a statistical perspective, the model fits the data well, and outperforms the NU mixture model of Dean and Raftery (2005) that was proposed for gene expression analysis. To get a rough idea on how the GNG model would perform for gene expression data and to further compare with the NU model for such data, the GNG model was used to fit the gene expression data analyzed in Dean and Raftery (2005). In this preliminary study, the genes that were differentially expressed according to the NU mixture model were similarly classified by the GNG model (data not shown), although differences exist in some cases. These initial findings warrant further inquiry into the general applicability of the GNG model for classifying data obtained from various types of two-color arrays, but it is not within the scope of the current paper.

Through a bootstrap resampling technique, we evaluated the reliability of the loci that are flagged as hypermethylated. For all three cell lines, the call consistency was at least close to 95% based on *B* = 200 bootstrap samples, which provided us with high confidence in the loci labeled as hypermethylated. As discussed earlier regarding making inferences on classifying a probe as hypermethylated, we suggested setting the strength-of-evidence constant *C* to be inversely proportional to the ‘reliability’ of the microarray. Such a priori information may be learned through a standard known test set. However, if such information is not readily available, then the bootstrap procedure might be applied to various constants *C*’s. The one that would lead to a high consistency rate could then be chosen for making inferences.

Four lines of evidence were sought to evaluate the biological relevance of the loci identified as hypermethylated among the three cell lines: the number of singularly and commonly (both two ways and three ways) methylated loci; the gene ontology analysis of the commonly methylated loci in all three cell lines; the experimental verification of a subset of the flagged loci using methylation-specific PCR; and the literature supports of key methylated loci identified. Since the three cell lines are all estrogen receptor-positive with key similarities, but there are also clear differences in their phenotypic characteristics, the results match well with such biological knowledge. The GNG model revealed that MCF7 and T47D cell lines had similar amount of hypermethylated genes whereas the cell line MDA-MB-361 had significantly less methylated targets. At this global level, the GNG model portrays a relationship between DNA methylation and aging. A more aggressive tumor increases in severity quickly (such as MDA-MB-361) thereby progresses with little accumulation of methylation marks. Tumors from older patients (such as MCF7) develop less aggressively thereby accumulate more methylation events along the way. With the advent of microarray technology (current microarray platform interrogates close to 11,000 gene promoters), the GNG model uncovers many methylation events previously not described in the literature. We chose to validate a small set of genes identified as hypermethylated in MCF7. These genes had varying degree of methylation in comparison to immortalized normal breast epithelial cell line MCF10A. This experimental outcome in part substantiates the usefulness of the GNG model. A survey of the literature uncovers gene targets methylated concurrently or in a subset of the three breast cancer cell lines studied as described by other researchers. Methylation profiles of some of these genes are highlighted (not exhaustively) to provide additional support for these gene targets (black bullets in figure 2D).

It would also be of interest to evaluate the biological relevance of the methylated genes through its cross-correlation with gene expression data. To this end, we briefly examined a few of the genes that are commonly assayed in both the Affymetrix gene expression array based on which the gene expression data on the same cell lines were generated and the 44 K methylation array. It appears that some of the genes that are hypermethylated are indeed down-regulated in their gene expression, but given the small number of genes examined, it is diffcult to draw any general conclusion at this point. However, it would be of great interest to find the genes that are both hypermethylated and down-regulated, as they might be potential biomarkers for cancer diagnosis, prognosis, or even targets of demethylation drugs.

Finally, we note that although the GNG model was proposed for analyzing single slide methylation data due to the need for such a procedure, the formulation can be easily ex tended to the analysis of multi-slide data. One possibility would simply be using the average normalized log ratios, following Dean and Raftery (2005). Another would use normalized (within and between slides) log ratios from each slide directly in the joint likelihood formulation to increase the sample size and hence accuracy of the parameter estimates. Be it as a single-, or a multi-slide analysis tool, the GNG model can be used for data from other microarray platforms, such as gene expression data.

## Figures and Tables

**Table 1.** Parameter Estimates in the Three Experiments.

**Estimates**	**MCF7**	**T47D**	**MDAMB361**
(α̂_1_, β̂_1_)	(0.94, 0.42)	(1.07, 0.31)	(0.95, 0.37)
(μ̂, σ̂)	(0.002, 0.24)	(−0.01, 0.21)	(0.01, 0.20)
(α̂_2_, β̂_2_)	(1.11, 0.31)	(0.93, 0.36)	(0.91, 0.24)
(π̂_1_, π̂_2_, π̂_3_)	(0.16, 0.68, 0.16)	(0.13, 0.73, 0.15)	(0.16, 0.71, 0.14)

**Table 2.** Kullback-Leibler Distance Between the Fitted Models (GNG or NU) and the Ob served Data.

K-L Distance	MCF7	T47D	MDAMB361
KL(GNG, Obs.)	0.00055	0.00047	0.00086
KL(UN, Obs.)	0.022	0.017	0.026
(KL(UN, Obs.) − KL(GNG, Obs.))/KL(UN, Obs.)	0.97	0.97	0.97
